# Engineering Properties and Microstructure of Soils Stabilized by Red-Mud-Based Cementitious Material

**DOI:** 10.3390/ma17102340

**Published:** 2024-05-14

**Authors:** Wentao Li, Ke Huang, Feng Chen, Lihua Li, Yang Cheng, Kang Yang

**Affiliations:** 1 Key Laboratory of Health Intelligent Perception and Ecological Restoration of River and Lake, Ministry of Education, Hubei University of Technology, Wuhan 430068, China; wli20201027@hbut.edu.cn (W.L.); 102110914@hbut.edu.cn (K.H.); 102210900@hbut.edu.cn (Y.C.); 102110913@hbut.edu.cn (K.Y.); 2 School of Civil Engineering, Architecture and Environment, Hubei University of Technology, Wuhan 430068, China; 102010781@hbut.edu.cn

**Keywords:** red mud, magnesium oxide, calcium oxide, stabilized soil, freeze–thaw

## Abstract

Red mud (RM) is an industrial waste generated in the process of aluminum refinement. The recycling and reusing of RM have become urgent problems to be solved. To explore the feasibility of using RM in geotechnical engineering, this study combined magnesium oxide (MgO) (or calcium oxide (CaO)) with RM as an RM-based binder, which was then used to stabilize the soil. The physical, mechanical, and micro-structural properties of the stabilized soil were investigated. As the content of MgO or CaO in the mixture increased, the unconfined compressive strength (UCS) of the RM-based cementitious materials first increased and then decreased. For the soils stabilized with RM–MgO or RM–CaO, the UCS increased and then decreased, reaching a maximum at RM:MgO = 5:5 or RM:CaO = 8:2. The addition of sodium hydroxide (NaOH) promoted the hydration reaction. The UCS enhancement ranged from 8.09% to 66.67% for the RM–MgO stabilized soils and 204.6% to 346.6% for the RM–CaO stabilized soils. The optimum ratio of the RM–MgO stabilized soil (with NaOH) was 2:8, while that of the RM–CaO stabilized soil (with NaOH) was 4:6. Freeze–thaw cycles reduced the UCS of the stabilized soil, but the resistance of the stabilized soil to freeze–thaw erosion was significantly improved by the addition of RM–MgO or RM–CaO, and the soil stabilized with RM–MgO had better freeze–thaw resistance than that with RM–CaO. The hydrated magnesium silicate generated by the RM–MgO stabilized soil and the hydrated calcium silicate generated by the RM–CaO stabilized soil helped to improve the UCS of the stabilized soil. The freeze–thaw cycles did not weaken the formation of hydration products in the stabilized soil but could result in physical damage to the stabilized soils. The decrease in the UCS of the stabilized soil was mainly due to physical damage.

## 1. Introduction

Red mud (RM) is an industrial waste produced in the process of aluminum refinement. When 1 ton of alumina is produced, 1–1.5 tons of RM could be generated [1,2], and the annual production of RM worldwide could reach 120 million tons [3]. However, it is difficult for RM to be recycled and reused due to its high alkalinity [4,5], resulting in the consumption of land space, so it has to be disposed. Currently, the disposal storage of RM in China had reached more than one billion tons [6], which causes environmental pollution, groundwater pollution, and land salinization [7]. The recycling and reusing of RM have become urgent problems to be solved and have attracted lots of attention.

RM can be recycled and reused by recovering valuable metals, producing building materials [8], etc. Chavan et al. [9] used RM to partially replace cement to prepare concrete. Their results showed that when RM was used to replace 12% of the cement, the mechanical properties of the concrete were significantly improved. However, as the content of RM increased, the fluidity of the concrete decreased continuously. Some scholars [10] tried to use lime to stabilize kaolin, and after ten years, its UCS value was 21 times that of untreated kaolin. Magnesium oxide and sodium bicarbonate were used to stabilize kaolin and resulted in better strength measurements [11]. Recently, the application of RM as a road base material has emerged as a new way to recycle and reuse RM. Alam et al. [12] attempted to stabilize RM using Na_2_SiO_3_ and granulated ground blast furnace slag, and Sahile [13] added cement and fly ash. Their results all showed that the unconfined compressive strength (UCS) of stabilized RM was significantly improved and that it had potential as a road base material. Mukiza et al. [14] tried to apply RM in road engineering and found that RM alone had a low UCS. It was found that an amended RM performed better than natural soil as a road base material. These studies all regarded modified RM as a soil material and used it extensively in road engineering.

However, using a large content of RM in road engineering would have a negative impact on the surrounding environment [15,16,17]. Vigneshwaran et al. [18] found that increasing the content of RM had a negative effect, and RM had a better binding ability with sisal fiber when the content was 10–20%. RM contains active substances, such as SiO_2_ and Al_2_O_3_, that are good raw materials for synthesizing cementitious materials [19]. Zhang et al. [20] mixed RM with slag to make cementitious materials, which produced better mechanical properties. Sreelekshmi et al. [21] used RM, fly ash, and slag as raw materials to prepare cementitious materials. Their research results showed that the RM–fly ash–slag cementitious materials had good processability and strength characteristics. Therefore, using RM as a cementitious material to stabilize poor soil is another way to recycle and reuse RM. A two-year study found that the addition of 5% by weight of RM did not negatively affect plants and soil microorganisms, which indicated that RM could be used as a soil binder to stabilize soils [22]. To explore whether RM could replace more soil, it was found that RM mixed with 2% lime showed a greater strength in 20% of the soil than in 15% of the soil, which ensured that more soil could be replaced by RM after being treated with lime [23]. Some scholars have mixed RM with lime and then used it to stabilize expansive soils. It was found to improve the geotechnical properties of the soil [24]. Ma et al. [25] reported that the addition of an appropriate content of RM could effectively improve the UCS of stabilized soil. Suo et al. [26] investigated the mechanical properties of heavy-metal-contaminated soil stabilized by RM and desulfurized gypsum and found that after adding a stabilizer, the UCS of the contaminated soil increased by about 130–260%. In addition, in practice, temperature (i.e., freeze–thaw) is one of the main factors causing a deterioration in the performance of road base material [27,28,29,30]. However, few studies have investigated the capability of RM-based cementitious material (or binder) stabilized soil to resist freeze–thaw cycles.

Therefore, this study used magnesium oxide (MgO) (or calcium oxide (CaO)) to activate RM and then applied RM–MgO (or RM–CaO) to treat soil. The mechanical properties of RM-based cementitious materials were investigated, and the influence of freeze–thaw cycles on the physical and mechanical properties as well as the microstructure of alkali-activated RM-based cementitious material stabilized soil was explored. Nuclear magnetic resonance (NMR) tests, X-ray diffraction (XRD) tests, and scanning electron microscopy (SEM) were used to reveal the stabilization mechanism of the soil treated with the RM-based materials under freeze–thaw cycles.

## 2. Experimental Materials and Methods

### 2.1. Experimental Materials

**Figure 1:**
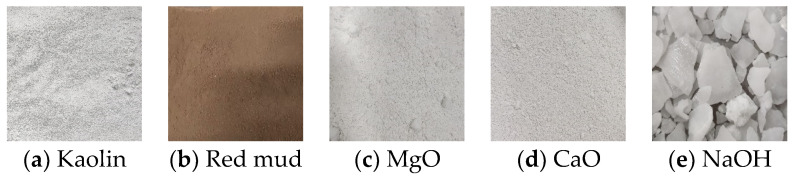
Experimental materials.

The plastic limit and liquid limit of the kaolin used in this study were 34.6% and 53.6%, respectively. It was provided by Guangzhou Chuangke New Material Technology Co., Ltd. (Guangzhou, China). RM was provided by Henan Porun New Material Co., Ltd. (Gongyi, China). Before testing, RM was dried in a 105 °C oven and passed through a 2 mm sieve. MgO was provided by Hebei Zhiyan Alloy Material Co., Ltd. (Xingtai, China), with an effective MgO content of >75%. CaO was provided by Gongyi Hengnuo Filter Material Co., Ltd. (Gongyi, China), with an effective CaO content of >90%. Sodium hydroxide (NaOH) was provided by Hebei Zhiyan Alloy Material Co., Ltd. (Xingtai, China), with a purity of >99%. Table 1 provides the chemical composition of the test soil and the chemical binders used in this study. Photographs of the experimental materials are shown in Figure 1.

### 2.2. Experimental Design

**Figure 2:**
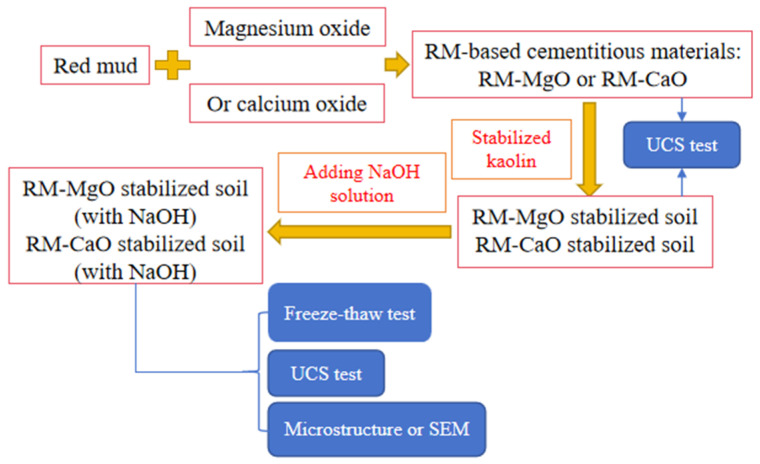
Flowchart.

A flowchart (Figure 2) has been inserted in this study to better show our methodology. Red mud is first combined with magnesium oxide (or calcium oxide) to form a cementitious material, which is then used to stabilize kaolin. Specimens of both the cementitious material and the stabilized soil were used for UCS testing. Finally, with the addition of NaOH solution to the stabilizer, the stabilized soil (with NaOH) was used for freeze–thaw testing, UCS testing, and microstructure or SEM testing.

#### 2.2.1. Mix Design for RM-Based Cementitious Material

RM and MgO (or CaO) were blended with water. After blending them evenly, the mixture was placed into a cubic mold with a length × width × height of 50 mm × 50 mm × 50 mm and stood for 6 h before demolding. After demolding, the specimens were sealed and placed in a standard curing box (with a temperature of 20 °C ± 2 °C and relative humidity of >95%) for curing. Table 2 shows the testing scheme for the experiment:

#### 2.2.2. Soil Stabilization with RM-Based Cementitious Material

To improve the UCS of the specimens, NaOH was added to RM–MgO and RM–CaO stabilized soil [31,32]. As comparison, RM–MgO and RM–CaO without NaOH were used to stabilize the soil. Cylindrical specimens with a size of Φ50 mm × 100 mm were prepared by compaction method. Firstly, RM, MgO (or CaO), kaolin, and water were mixed according to the design mix ratio. After stirring them evenly, compaction was carried out by using a 0.51 kg hammer, which fell freely along a steel bar that was 270 mm long. The mixture was divided into three layers and placed into the mold. Each layer was compacted 30 times with a unit compaction energy of 606.72 kJ × m^−3^, which is slightly higher than the 605.6 kJ × m^−3^ required by the ASTM standard Proctor test [33]. After compaction was completed, the mold was removed using a jack, and the specimens were sealed with plastic wrap and placed in a standard curing box for curing until the design time. The UCS test was carried out using a universal testing machine with a range of 20 kN. The loading rate was controlled at 1 mm × min^−1^ [34]. The test was stopped immediately after the UCS reached its peak value. The test was performed twice. It was important to note that ASTM D2166M-16 [35] specifies that the specimen size for the UCS test should have a minimum diameter of 30 mm and maximum particle size less than one-tenth of the diameter of the specimen. The height-to-diameter ratio of the specimen should be between 2 and 2.5. In this study, the specimen size met these requirements. The compaction method used in this study was adopted for two reasons. First, it ensured that the size of the compacted specimen was consistent with the specimen size of the UCS test, which improved the accuracy of the test results. Second, it was necessary due to the limited test materials and test conditions in the laboratory.

**Figure 3:**
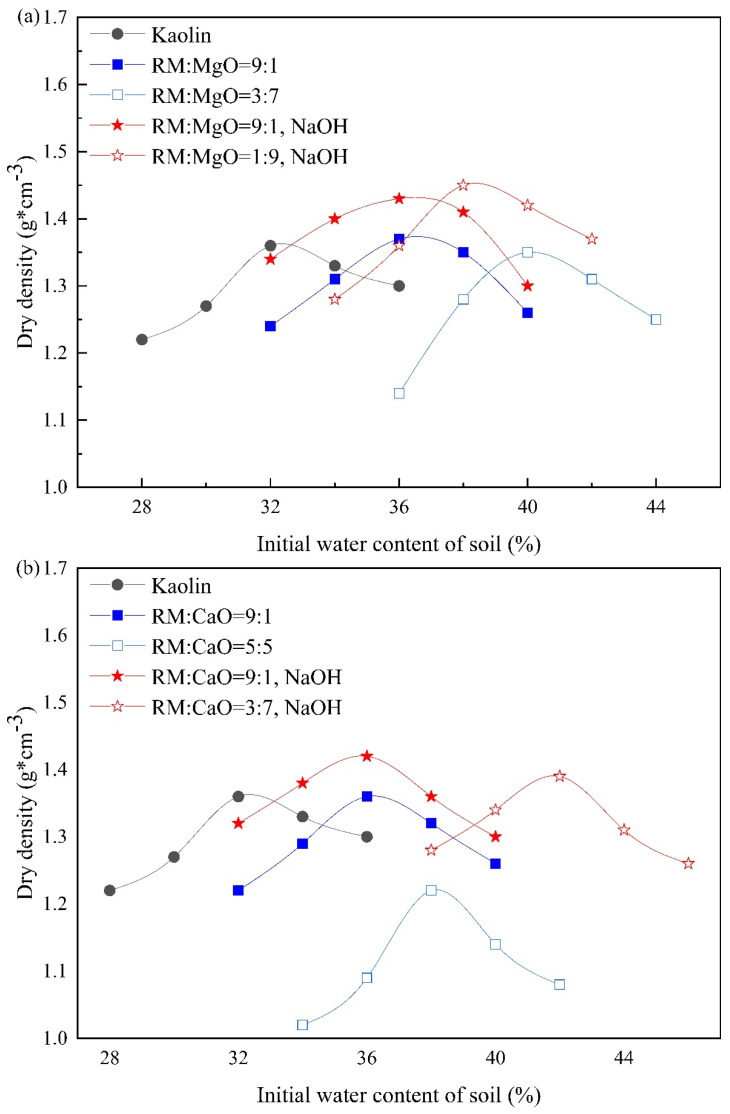
Compaction curves for stabilized soil: (**a**) RM–MgO (or NaOH) stabilized soil; (**b**) RM–CaO (or NaOH) stabilized soil.

Figure 3 provides the compaction curve of RM-based cementitious material stabilized soil. As the MgO (or CaO) content increased, that is, as the content of RM in the stabilizer decreased, the optimum moisture content (OMC) of RM–MgO stabilized soil (with or without NaOH) increased. For example, without NaOH solution, as the OMC for the soil stabilized at ratios of RM:MgO = 8:2, 7:3, 6:4, 5:5, and 4:6 was expected to be between the two OMCs at ratios of RM:MgO = 9:1 and 3:7. Compaction tests for the ratios of 8:2, 7:3, 6:4, 5:5, and 4:6 were not conducted. The average OMC values that were obtained from soils at ratios of RM:MgO = 9:1 and 3:7 were used to prepare UCS specimens stabilized at ratios of 8:2, 7:3, 6:4, 5:5, and 4:6. Therefore, the OMC values of RM–MgO (with or without NaOH) stabilized soil were 37% and 38%, respectively. The OMC values of RM–CaO (with or without NaOH) stabilized soil were 39% and 37%, respectively.

Similar to previous research [36], the content of stabilizer was 15% (by the weight of dry soil). The mass ratios of RM, MgO, and CaO in the stabilizer are shown in Table 3.

#### 2.2.3. Freeze–Thaw Test

According to the reference [37], the freezing temperature was set to −20 °C and the thawing temperature to 20 °C based on the temperature range in China. A complete freeze–thaw cycle consisted of 12 h of freezing and 12 h of thawing. After the specimen was maintained to the specified curing time, it would be vacuumed and saturated in the vacuum saturator. To prevent evaporation of water from the specimens during the freeze–thaw process, they were wrapped in plastic wrap and placed in a test chamber. When the specimens reached the required cycles (0, 3, 6, and 10 times), their mass and volume were measured, which were then used for calculating the mass loss rate and volume change.

(1) Equation (1) was used to calculate the mass loss rate of RM–MgO (or RM–CaO) stabilized soil.

(1) $$\Delta M=(M_{0}-M_{i})/M_{0}\times 100\%$$

where ΔM—the mass loss rate of stabilized soil after *i* freeze–thaw cycles (%), *i*—the cycle number, $M_{0}$—the initial mass of stabilized soil, and $M_{i}$—the mass of stabilized soil after *i* freeze–thaw cycles.

(2) By measuring the height and diameter of each specimen, the volume of the specimen before and after freeze–thawing was obtained. The method used to calculate the volume change rate of RM–MgO (or RM–CaO) stabilized soil is shown in Equation (2):

(2) $$\Delta V=(V_{0}-V_{i})/V_{0}\times 100\%$$

where ΔV—the volume change rate of stabilized soil after *i* freeze–thaw cycles (%), *i*—the cycle number for which a positive value indicates volume shrinkage and a negative value indicates volume expansion, $V_{0}$—the initial volume of stabilized soil, and $V_{i}$—the volume of stabilized soil after *i* freeze–thaw cycles.

#### 2.2.4. Microstructure or SEM

After the UCS test was completed, block soil samples were collected and dried in a freeze dryer. The dried samples were ground and passed through a 0.075 mm sieve. The sieved samples were used for XRD tests using a Panalytical Empyrean instrument (Panaco, Almelo, The Netherlands) with a scanning rate of 5°/min. Some unsieved samples were subjected to SEM tests using a Hitachi SU8010 instrument (Hitachi High-Tech, Tokyo, Japan) with resolution of 1 nm/15 kv and 1.3 nm/1 kv. In addition, crushed soil samples with a diameter of 10–15 mm and a height of 20–30 mm were saturated using a vacuum pump. These saturated samples were then used in NMR tests. A PQ-001-Mini nuclear magnetic resonance instrument (Shanghai Newmax Electronic Technology Co., Shanghai, China) with a resonance frequency of 12 MHz, a magnetic induction intensity of 0.52 T, and an effective test range of 60 mm × Φ60 mm was used.

## 3. Results and Discussion

### 3.1. UCS of RM-Based Cementitious Material

**Figure 4:**
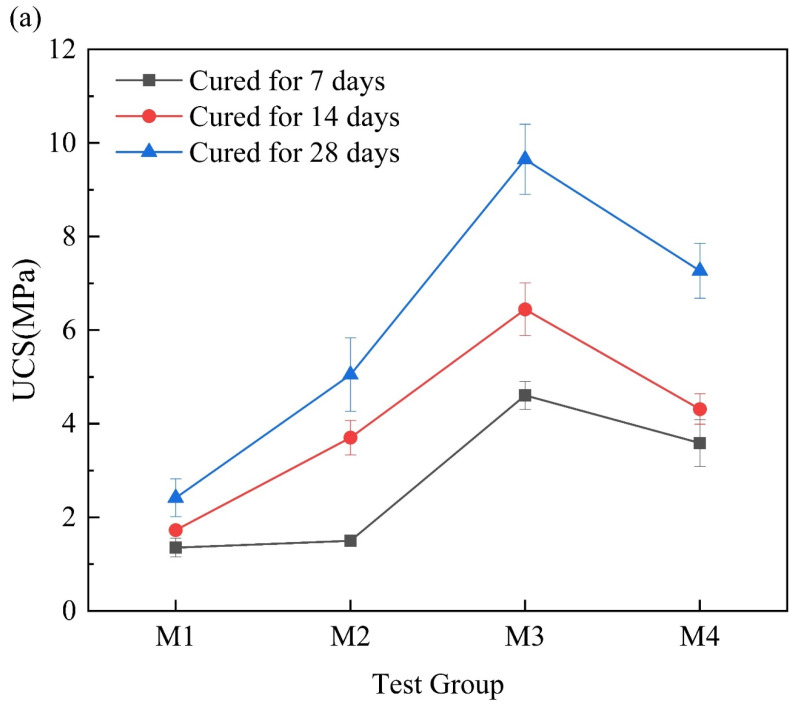

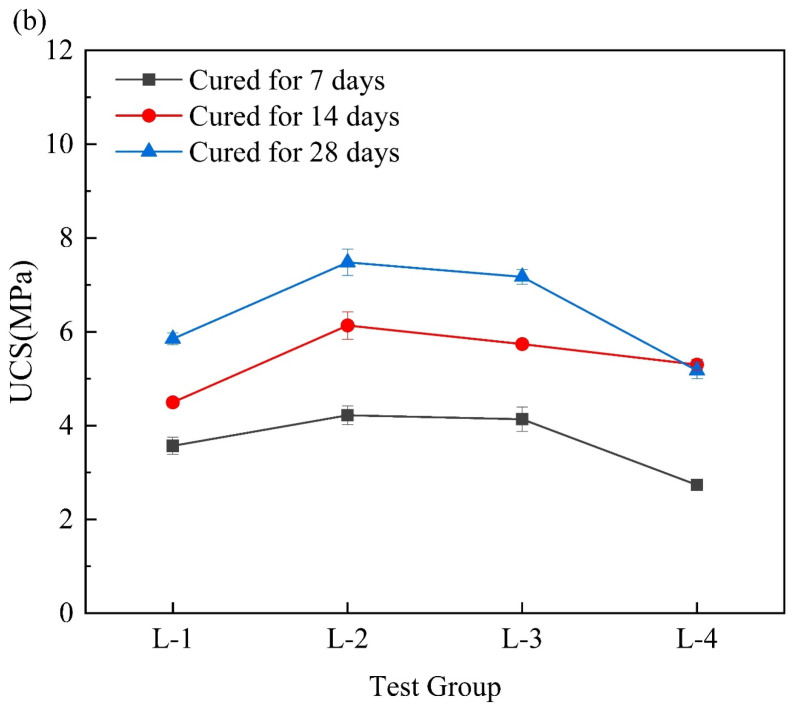
(**a**) UCS of RM–MgO cementitious material; (**b**) UCS of RM–CaO cementitious material.

Figure 4a shows the UCS of the MgO-activated RM cementitious material. As the content of MgO in the mixture increased, the UCS first increased and then decreased. The optimal ratio was RM:MgO = 3:4, with a 7-day UCS of 4.60 MPa. Figure 4b shows the UCS of the CaO-activated RM cementitious material. As the content of CaO increased, the UCS also first increased and then decreased, with the optimal ratio of RM:CaO = 3:2 and a 7-day UCS of 4.22 MPa. This indicated that both MgO and CaO had a significant activation effect on the RM, and under their respective optimal ratios, the UCS of the RM–MgO cementitious material was 5–29% higher than that of the RM–CaO cementitious material. According to Nie et al. [38], it was found that the 7-day UCS of the RM–fly ash cementitious material reached about 2.5 MPa, and the 28-day UCS was only about 3 MPa, both of which were lower than the UCS when the optimal ratio was used in this experiment. This illustrated that MgO and CaO had better effect on the activation of the RM.

### 3.2. UCS of Stabilized Soil

The above results indicated that the RM-based cementitious materials had the potential to stabilize the soil. Therefore, further tests were carried out on the physical and mechanical properties of the RM-based cementitious material stabilized soil.

#### 3.2.1. UCS of RM–MgO (or RM–CaO) Stabilized Soil without NaOH

**Figure 5:**
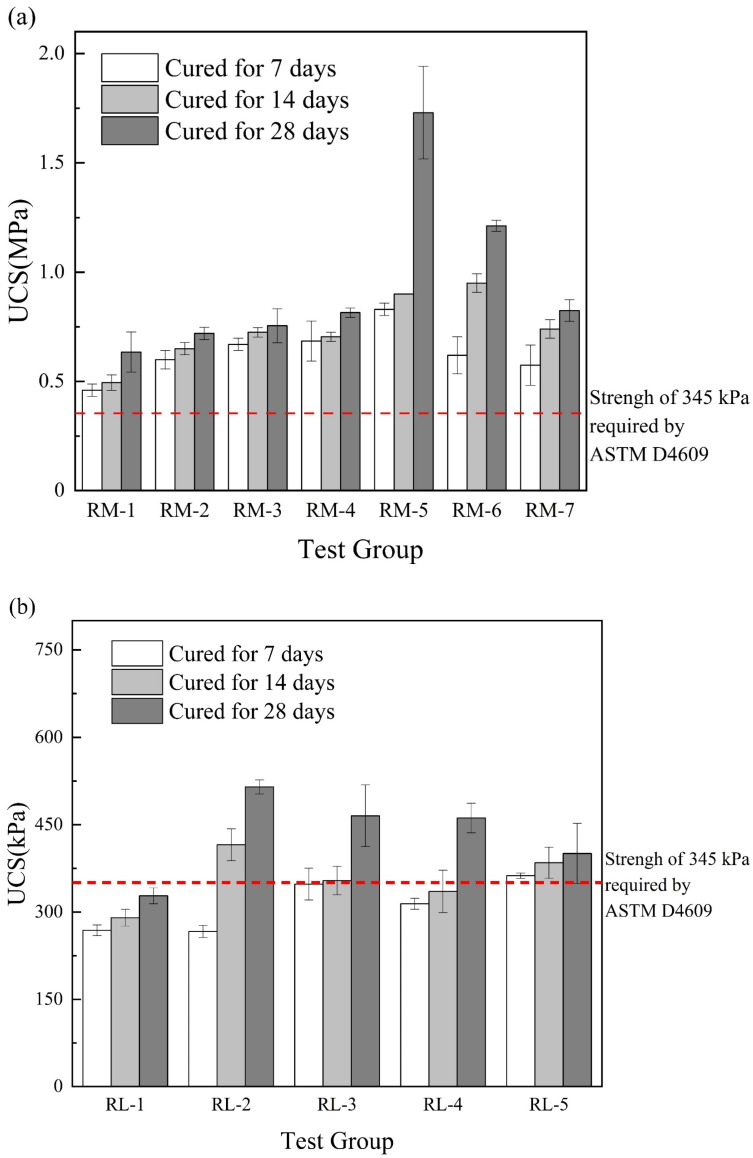
(**a**) UCS of RM–MgO stabilized soil (without NaOH); (**b**) RM–CaO stabilized soil’s UCS (without NaOH) [41].

Figure 5a shows the change in the UCS of the RM–MgO stabilized soil. The UCS of the stabilized soil first increased and then decreased as the content of RM decreased. The 7-day and 14-day UCS values of the stabilized soils were most significantly enhanced when the RM content was 50–60%. At 28 days, the RM-5 (RM:MgO = 5:5) reached a maximum UCS of 1.73 MPa. Figure 5b shows the relationship between the UCS and the content of RM for the RM–CaO stabilized soils. After 7 days of curing, as the content of the RM in the mixture decreased, the UCS gradually increased, and when the mass ratio of the RM to CaO was 5:5, the UCS was optimal. However, when the curing times were 14 and 28 days, the UCS of stabilized soil first increased and then decreased as the content of the RM in the mixture decreased. At this time, when the mass ratio of RM to CaO was 8:2, the maximum UCS was obtained. This might be because the RM activity was not fully activated when the MgO (or CaO) content was low. As the content of MgO (or CaO) increased, its activity was fully activated to reach the maximum UCS value. However, when the optimum content of MgO (or CaO) was exceeded, the MgO (or CaO) would be dispersed in the soil in its natural state, which would weaken the connection between the soil and lead to a reduction in UCS [39,40]. ASTM [41] advised that the UCS of admixtures for improving the engineering properties of fine-grained soil should not be less than 345 kPa. For the RM–CaO stabilized soil, only the 7-day UCS for RL-5 (RM:CaO = 5:5) met this requirement, only the 14-day UCS for RL-5 and RL-2 (RM:CaO = 5:5 and 8:2) met this requirement. Additionally, the 28-day UCS of RL-2, RL-3, RL-4, and RL-5 (RM:CaO = 8:2, 7:3, 6:4, and 5:5) met this requirement. Conversely, the UCS of the RM–MgO stabilized soil met this requirement at all curing times. Overall, the UCS of the RM–CaO stabilized soil was lower than that of the RM–MgO stabilized soil.

#### 3.2.2. UCS of RM–MgO (or RM–CaO) Stabilized Soil with NaOH

**Figure 6:**
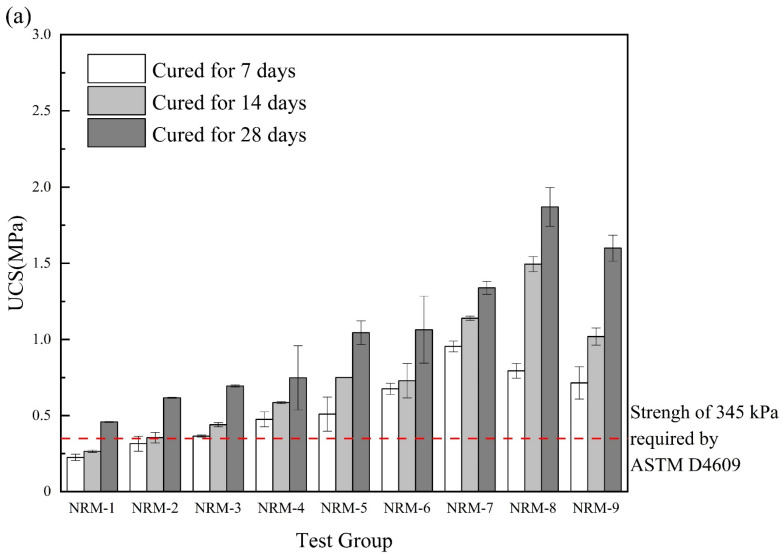

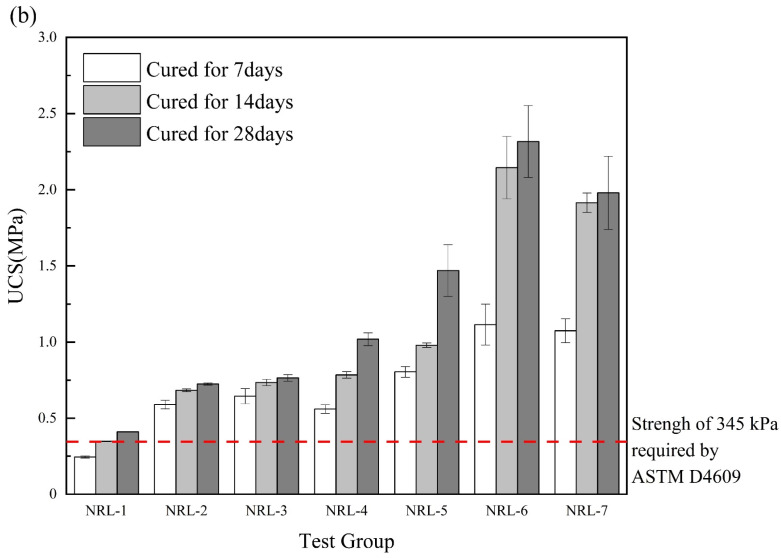
(**a**) UCS of RM–MgO stabilized soil (with NaOH); (**b**) RM–CaO stabilized soil’s UCS (with NaOH) [41].

As shown in Figure 6a, the UCS of the RM–MgO stabilized soil increased after adding NaOH. With the decrease in RM content, the UCS showed a trend of increasing and then decreasing, and the maximum 7-day UCS was obtained when the RM content was 30%, while the maximum 14-day and 28-day UCS were obtained when the RM content was 20%. As seen in Figure 6b, for the RM–CaO stabilized soil, the UCS of the soil with NaOH was much higher than that of the stabilized soil without NaOH. As the content of RM continued to decrease, the UCS of the stabilized soil showed a trend of first increasing and then decreasing. When the mass ratio of RM to CaO was 4:6, the maximum UCS of the stabilized soil was obtained. Although RM–MgO (or RM–CaO) could accelerate the UCS development of stabilized soils, excessive residual MgO (or CaO) negatively affected the UCS of the stabilized soils. The optimal ratios for the stabilized soil with NaOH (RM:MgO = 2:8, RM:CaO = 4:6) require more MgO (or CaO) than the optimal ratios for the stabilized soil without NaOH (RM:MgO = 5:5, RM:CaO = 8:2), probably because the addition of NaOH requires more MgO (or CaO) to participate in the hydration reaction, which continues to increase in UCS. Equation (3) was used to calculate the rate of the UCS enhancement of the soil stabilized with RM–MgO (or RM–CaO). The UCS enhancement rate of the RM–MgO stabilized soil ranged from 8.09% to 66.67%, while the UCS enhancement rate of the RM–CaO stabilized soil ranged from 204.6% to 346.6%. According to Cristelo et al. [31], the addition of NaOH accelerated the dissolution of silica and alumina, thereby producing more hydration products to increase the UCS of the RM–MgO (or RM–CaO) stabilized soil. The UCS of all specimens generally met the requirements of ASTM [41] specifications.

(3) $$\Delta S=(S_{1}-S_{0})/S_{0}\times 100\%$$

where ΔS—the rate of the UCS enhancement of the stabilized soil, $S_{1}$—the UCS of the stabilized soil with NaOH, and $S_{0}$—the UCS of the stabilized soil without NaOH.

### 3.3. Physical, Mechanical, and Microstructural Charateristics of Stabilized Soil under Freeze–Thaw Cycles

The specimens with the optimal ratios (NRM-8 and NRL-6) determined in Section 3.2 were subjected to freeze–thaw cycles after being cured for 28 days. Their mass and volume were monitored. UCS, NMR, XRD, and SEM tests were performed on the NRM-8 and NRL-6 specimens, and then the performance was compared with pure MgO stabilized soil (NRM-0) and pure CaO stabilized soil (NRL-0).

#### 3.3.1. The Influence of Freeze–Thaw Cycles on Mass Change

**Figure 7:**
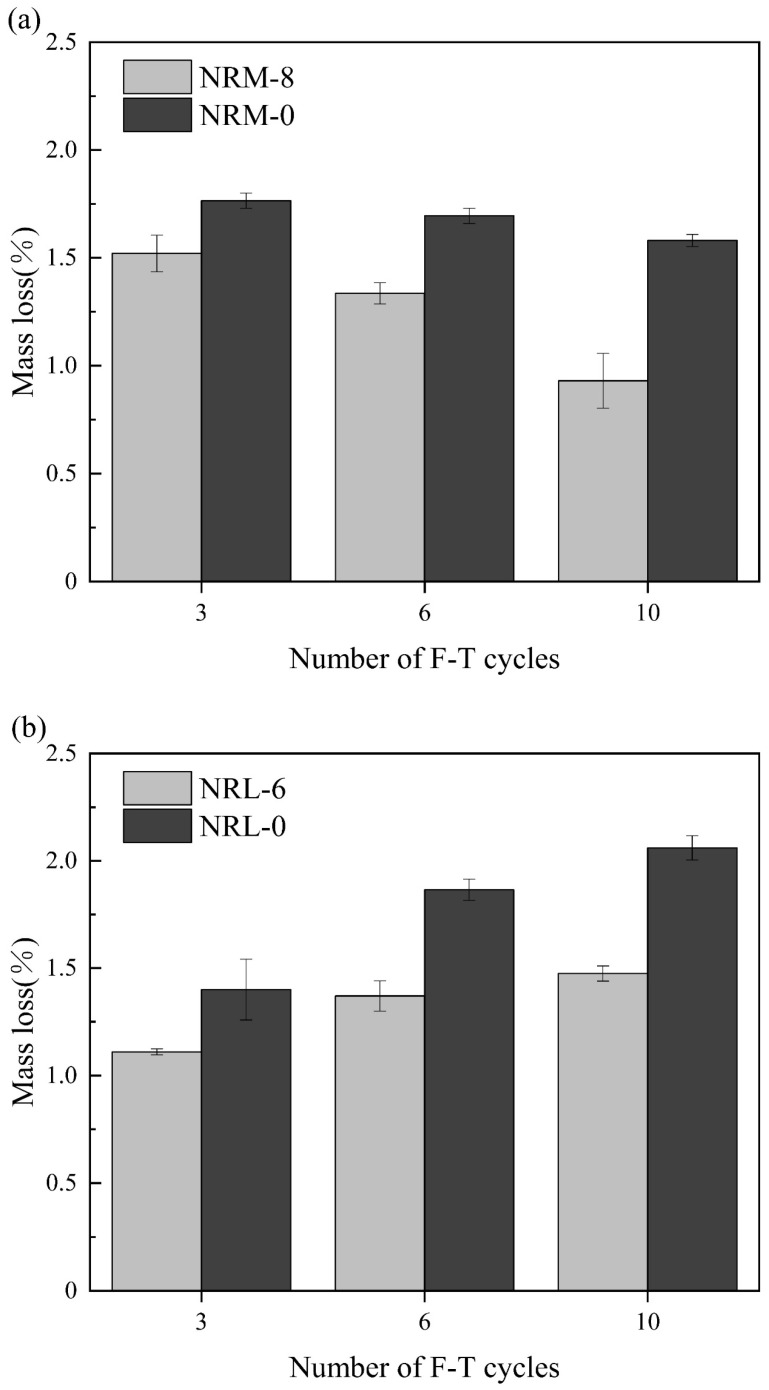
Mass loss rate during freeze–thaw cycles: (**a**) NRM-8, NRM-0; (**b**) NRL-6, NRL-0.

Figure 7 shows the mass loss rate of the stabilized soil with an increasing number of cycles. The mass loss rate of the RM–MgO stabilized soil decreased as the cycle number grew, while that of the RM–CaO stabilized soil increased. For the RM–MgO stabilized soil, as the cycle number grew from zero to three, the mass loss rate of NRM-8 and NRM-0 reached their maximum values of 1.52% and 1.77%, respectively. As the cycle number grew to six, the mass loss rate of NRM-8 started to decrease to 1.34%, while that of NRM-0 decreased to 1.70%. As the cycle number progressed from three to ten, the mass loss rate of NRM-8 decreased more than that of NRM-0. For the RM–CaO stabilized soil, when the cycle number progressed from zero to three, the mass loss rate increased to 1.11% for NRL-6 and 1.40% for NRL-0. As the cycle number progressed to six, the mass loss rate of NRL-6 continued to increase to 1.37% and that of NRL-0 increased to 1.87%, while the increase in the mass loss rate of NRL-6 was less than that of NRM-0. When the cycle number progressed from six to ten, the mass loss rate of NRL-6 continued to increase less than that of NRL-0. The mass loss rate of the RM–MgO stabilized soil (or RM–CaO stabilized soil) was always lower than that of the pure MgO stabilized soil (or the pure CaO stabilized soil) for the same cycle number. This indicated that for both stabilized soils, the addition of RM–MgO (or RM–CaO) can effectively prevent the mass loss caused by freeze–thaw cycles. As the cycle number increased from zero to ten, the mass loss of NRM-8 first increased and then decreased, while the mass loss rate of NRL-6 kept increasing, so the RM–MgO stabilized soil had better resistance to mass loss. The reason for this might be that the expansion stress caused by freezing led to internal cracks in the specimen. Then, more external water was transferred into the internal voids and cracks, thus decreasing the mass loss rate of the specimen. When the mass loss of the specimen due to spalling was higher than the mass gained by water absorption, the mass loss rate of the specimen increased [42,43].

#### 3.3.2. The Influence of Freeze–Thaw Cycles on Volume Change

**Figure 8:**
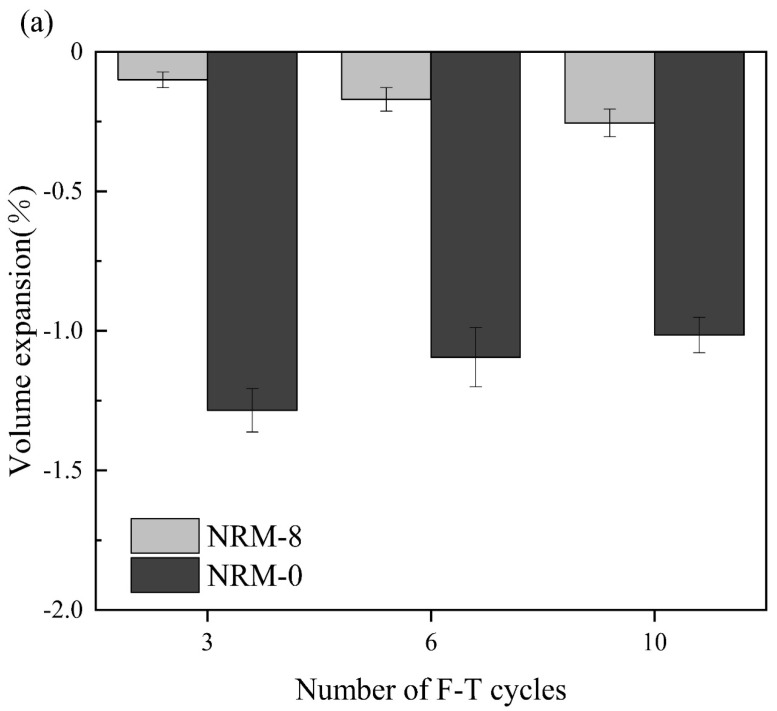

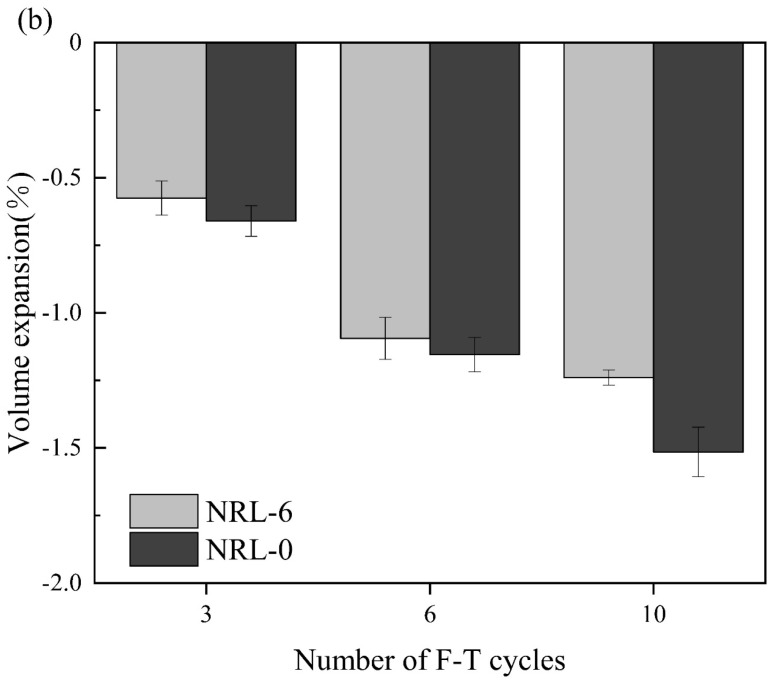
Volume expansion rate during freeze–thaw cycles: (**a**) NRM-8, NRM-0; (**b**) NRL-6, NRL-0.

Figure 8 shows the volume expansion rate of the stabilized soil with the number of cycles. The volume expansion rate of the RM–MgO stabilized soil increased slightly with the increase in cycle number. Conversely, the volume expansion rate of the RM–CaO stabilized soil showed an increasing trend with the cycle number. As the cycle number rose from zero to three in the RM–MgO stabilized soil, the volume expansion of NRM-8 increased to 0.10% and that of NRM-0 rose to 1.29%. This indicated that the volume expansion of freeze–thaw cycles was suppressed by the addition of RM–MgO. From cycles 3 to 10, the volume expansion of NRM-8 increased to 0.17% and the volume expansion of NRM-0 slightly decreased to 1.10%. Furthermore, NRM-8 showed much lower volume expansion compared to NRM-0. For the RM–CaO stabilized soil, when the cycle number was increased from zero to three, the volume expansion rate started to increase to 0.58% for NRL-6 and to 0.66% for NRL-0. As the cycle number rose to 10, the volume expansion rate of NRL-6 increased to 1.24% and that of NRL-0 increased to 1.52%. Additionally, the volume expansion rate of NRL-6 was always lower than that of NRM-0. The volume change rate of the RM–MgO stabilized soil (or the RM–CaO stabilized soil) was always lower than that of the pure MgO (or pure CaO) stabilized soil for the same cycle number. This indicated that for both stabilized soils, the addition of RM–MgO (or RM–CaO) could effectively prevent the volume expansion caused by freeze–thaw cycles. As the cycle number increased from zero to ten, the volume expansion rate of NRM-8 was much lower than that of NRL-6, indicating that the RM–MgO stabilized soil resisted the volume expansion better. According to the results of Konrad [44], the stabilized soil expanded because the temperature difference between the surface and the interior caused water to migrate from the inside to the outside. Some of the pores enlarged by freezing and melting water could not return to their original state, resulting in expansion.

#### 3.3.3. The Influence of Freeze–Thaw Cycles on UCS

**Figure 9:**
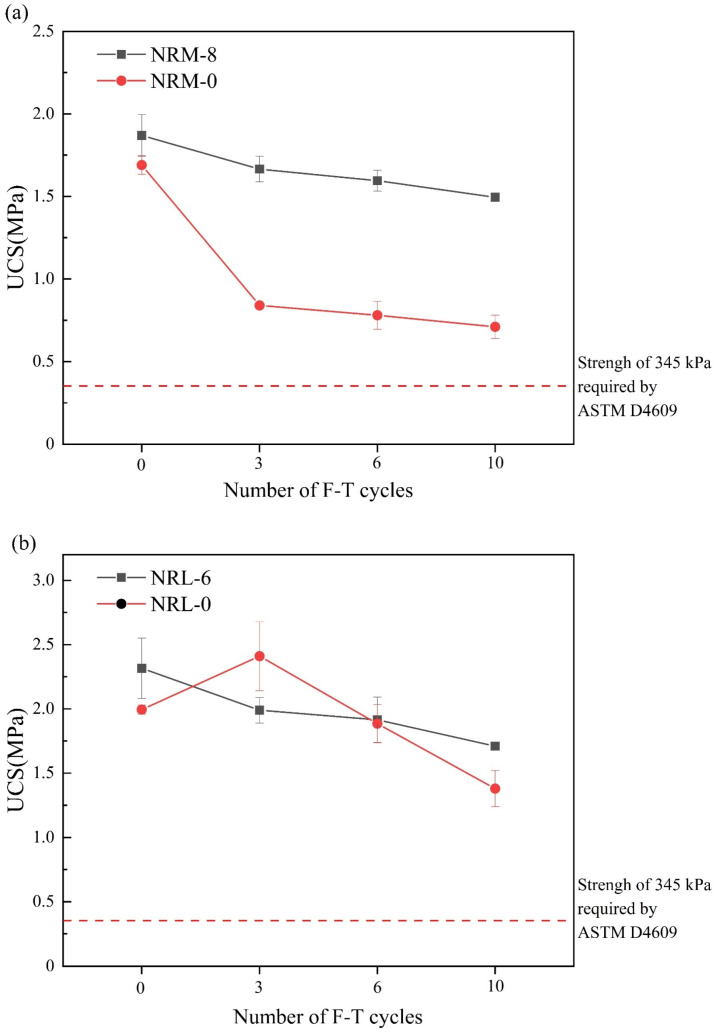
Change in UCS during freeze–thaw cycles: (**a**) NRM-8, NRM-0; (**b**) NRL-6, NRL-0 [41].

Figure 9 shows the UCS of the stabilized soil during freeze–thaw cycles. The UCS of the stabilized soil showed a decreasing trend as the cycle number increased. For the RM–MgO stabilized soil, when the cycle number increased from zero to three, the UCS of NRM-8 decreased from 1.87 MPa to 1.67 MPa, while the UCS of NRM-0 decreased from 1.69 MPa to 0.84 MPa. The UCS of NRM-8 decreased significantly more than that of NRM-0 (the UCS decrease was the ratio of the difference between the UCS after the freeze–thaw cycles and the UCS without freeze–thaw cycles). As the cycle number increased from three to ten, the UCS values of both NRM-8 and NRM-0 decreased. However, the UCS of NRM-8 was consistently higher than that of NRM-0. For the RM–CaO stabilized soil, when the cycle number increased from zero to ten, the UCS of NRL-6 slightly decreased, but that of NRL-0 first increased and then decreased. Yang et al. [30] found that freeze–thaw cycles destroyed the soil’s structure and reduced its strength. Nevertheless, they also triggered the migration of water, which stimulated the hydration of the binders. Consequently, the soil structure was strengthened again, showing first increased and then decreased UCS values. As the cycle number increased from zero to ten, the UCS of NRL-6 (or NRM-8) was always higher than that of NRL-0 (or NRM-0).

The UCS of the stabilized soil generally decreased with the increase in the cycle number, which indicated that freeze–thaw erosion still had a deteriorating effect on the UCS of the soil. The UCS of the soils decreased because the freezing of the water made them swell, and repeated incidents of swelling made the soils looser and weaker [45]. In addition, the UCS of the RM–MgO stabilized soil (or the RM–CaO stabilized soil) was significantly higher than that of the pure MgO stabilized soil (or the pure CaO stabilized soil) after 10 freeze–thaw cycles. The test results showed that for soils, the addition of RM–MgO (or RM–CaO) was effective in mitigating the loss of UCS caused by freeze–thaw erosion. Nevertheless, the UCS of the RM–MgO and RM–CaO stabilized soils during the freeze–thaw cycles still met ASTM [41] strength standards.

#### 3.3.4. NMR

**Figure 10:**
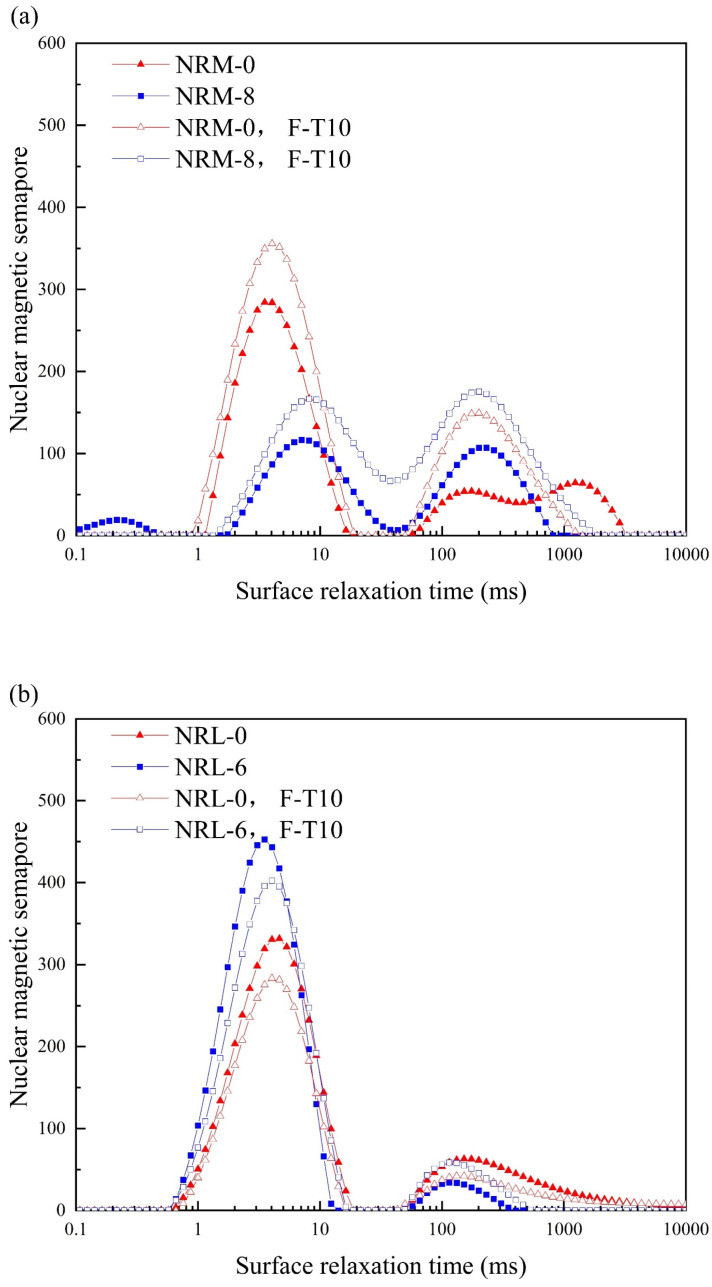
NMR before and after freeze–thaw cycles: (**a**) NRM-8, NRM-0 and (**b**) NRL-6, NRL-0. F-T10: after 10 freeze–thaw cycles.

To explore the influence of freeze–thaw cycles on the pore structure of the stabilized soil, NMR tests were used. In Figure 10, the main peaks of the specimens were mainly distributed between 0.1 ms and 10 ms and between 10 ms and 1000 ms according to transverse relaxation times. The transverse relaxation time was positively correlated with pore size. The two main peaks corresponding to pores were defined as small pores and large pores, respectively, and the peak area (i.e., the closed area formed by the T2 curve and the X-axis) represented the pore volume [46].

Figure 10 shows the T2 distribution curves of NRM-0, NRL-0, NRM-8, and NRL-6. From Figure 8a, the peak areas of both the small and large pores in NRM-8 and NRM-0 were increased after 10 freeze–thaw cycles. Figure 8b showed that the peak area of small pores in NRL-6 and NRL-0 decreased after 10 freeze–thaw cycles, while the peak area of large pores increased slightly. Despite the different trends in the peak areas of the large and small pores in the NRL-6, the overall peak areas were basically unchanged after 10 freeze–thaw cycles. This indicated that the freeze–thaw cycles caused the pore size of the small pores in NRL-6 to increase and eventually transformed the small pores into large pores. He et al. [47] observed this phenomenon and found that freeze–thaw cycles transformed small pores in the soil into large pores. It was found that freeze–thaw cycles resulted in an increased pore volume in the stabilized soil of RM–MgO (or RM–CaO), and the total pore volume of NRM-8 (or NRL-6) was more than that of NRM-0 (or NRL-0), which might also account for the difference in UCS. The appearance of ice crystals in the specimen after freezing increased the diameter of the pores in the specimen, and after melting, the pore diameter could not be restored to its original state, which led to an increase in pore volume [47,48].

#### 3.3.5. XRD

**Figure 11:**
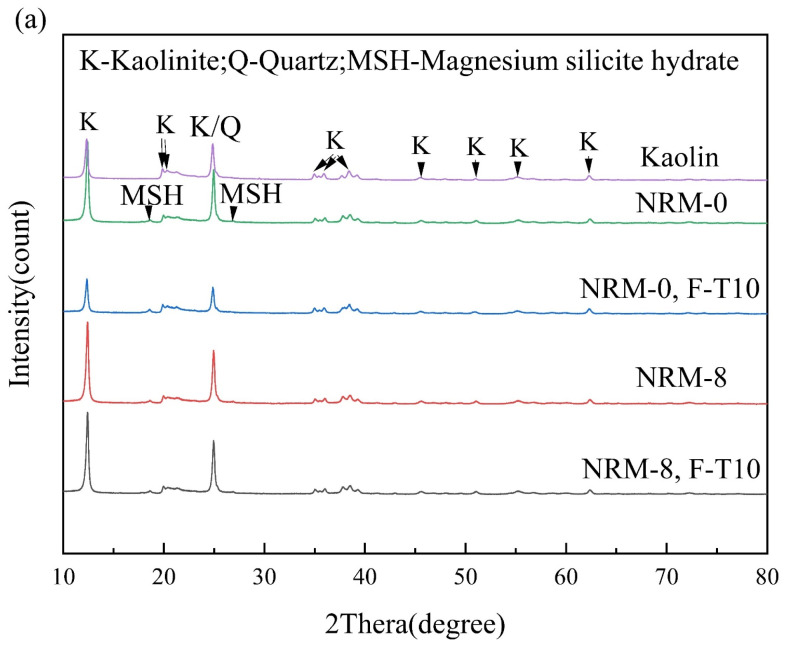

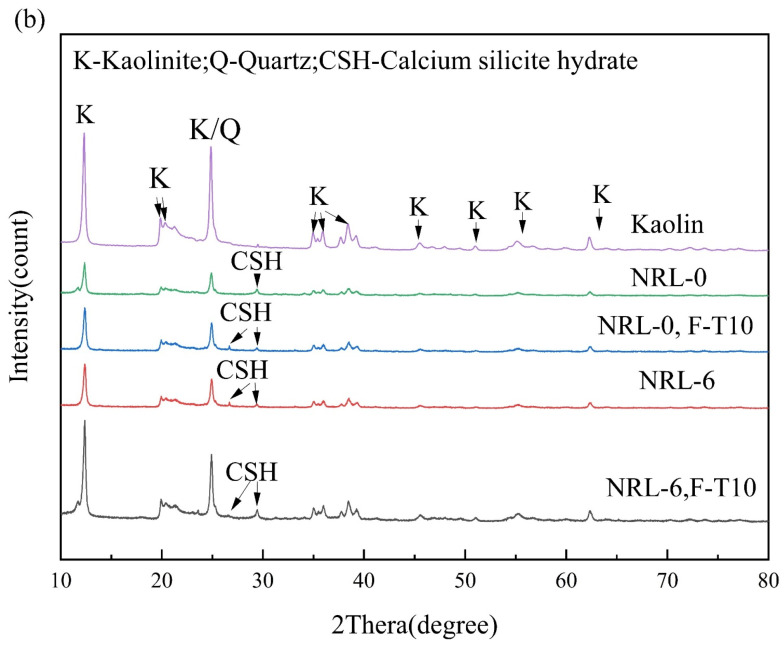
XRD before and after freeze–thaw cycles: (**a**) NRM-8, NRM-0 and (**b**) NRL-6, NRL-0. F-T10: after 10 freeze–thaw cycles.

Figure 11 provides the XRD results of the freeze–thaw cycle samples. After stabilization with RM–MgO (or RM–CaO), new diffraction peaks appeared in the samples compared to the original soil, indicating that new mineral components were generated in the soil after treatment. In the RM–MgO solidification system, the main hydration product generated was magnesium silicate hydrate (MSH), with diffraction peaks at 18° and 26°. In the RM–CaO solidification system, the main hydration product inside the soil was calcium silicate hydrate (CSH), whose diffraction peaks occurred at 26° and 28°. The production of MSH and CSH might be responsible for the increase in UCS [49]. The crystalline phases detected in the RM–CaO stabilized soil before and after the freeze–thaw cycles were similar, and so were those in the RM–MgO soil. Taking NRM-8 as an example, before the freeze–thaw cycles, MSH was detected at 18° and 26°. After the freeze–thaw cycles, the position and intensity of these diffraction peaks hardly changed. For the NRL-0 sample, before the freeze–thaw cycles, its CSH diffraction peaks were mainly observed at 28°. After 10 cycles, the intensity of this diffraction peak decreased, but a new CSH characteristic peak was detected at 26°. The above results indicated that the freeze–thaw cycles did not decrease the formation of hydration products (CSH or MSH), so the decrease in UCS of the RM–MgO (or RM–CaO) stabilized soil was mainly caused by physical damage.

#### 3.3.6. SEM

**Figure 12:**
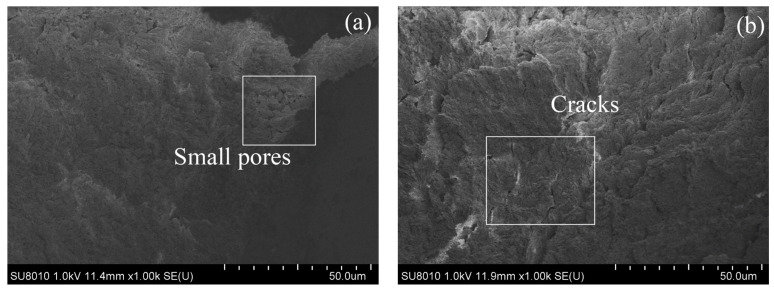

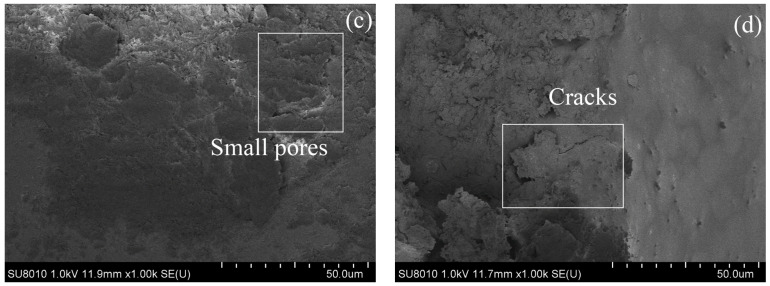
SEM of RM–MgO stabilized soil: (**a**) NRM-8, without freeze–thaw cycles; (**b**) NRM-8, after 10 freeze–thaw cycles; (**c**) NRM-0, without freeze–thaw cycles; (**d**) NRM-0, after 10 freeze–thaw cycles.

**Figure 13:**
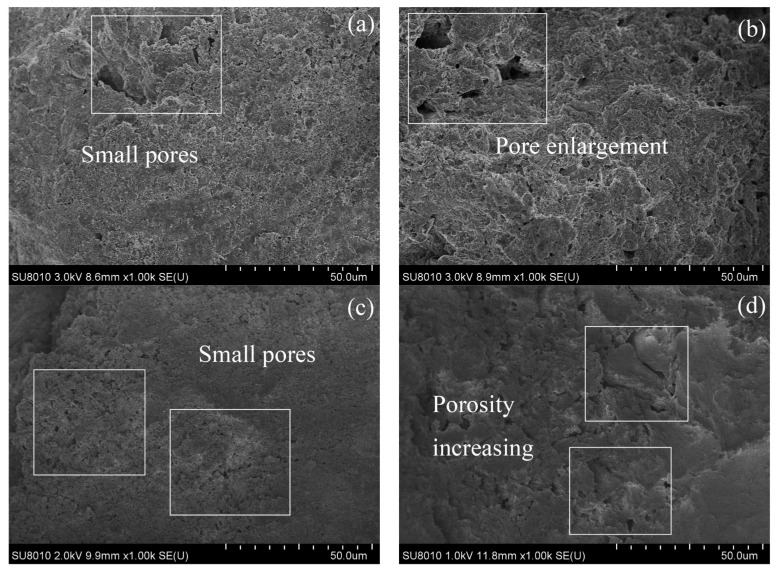
SEM of RM–CaO stabilized soil: (**a**) NRL-6, without freeze–thaw cycles; (**b**) NRL-6, after 10 freeze–thaw cycles; (**c**) NRL-0, without freeze–thaw cycles; (**d**) NRL-0, after 10 freeze–thaw cycles.

A representative image (magnified 1000 times) was selected for each sample in SEM tests, and the images are shown in Figure 12 and Figure 13. After 28 days of curing without freeze–thaw cycles, the soil particles of the RM–MgO stabilized soil (or the RM–CaO stabilized soil) made contact with each other, and only a few small pores were present. After 10 freeze–thaw cycles, fine cracks developed inside the RM–MgO stabilized soil, while the pore size for the RM–CaO stabilized soil increased significantly and its soil structure became looser. This indicated that freeze–thaw cycles reduced the particle density and bonding force in the RM–MgO (or RM–CaO) stabilized soil, resulting in damage to the soil structure and causing the UCS to decrease on a macroscopic level. The reason for this might be that repeated freeze–thaw cycles caused stress inside the soil, which caused a volume expansion in the samples and increased the pore volume in the soil [47]. As the pores increased in size and number, the distance between the pores decreased. Some pores connected or merged with each other, forming cracks.

## 4. Stabilization Mechanism

For the RM–MgO stabilized soil, when the RM, MgO, and water were mixed in the soil, the MgO in contact with the water produced a hydration reaction to form Mg(OH)_2_, as shown in Equation (4). As shown in Equations (5) and (6), some of the Mg(OH)_2_ dissociated in the water to form Mg^2+^ and OH^−^, which promoted the dissolution of reactive alumina and reactive silica in the RM. The dissolved silica and alumina were combined with Mg^2+^ in the soil to form MSH and other gelling substances. Similarly, for the RM–CaO stabilized soil, when the RM, CaO, and water were mixed in the soil, as in Equation (7), CaO generated Ca(OH)_2_ when it came into contact with the water. Part of the Ca(OH)_2_ would dissociate the Ca^2+^ and OH^−^ in the water, as shown in Equations (8) and (9), and the active alumina and active silicon oxide in the RM started to dissolve and polymerize with the free Ca^2+^ in the soil to generate gelling substances, such as CSH. MSH and CSH filled the pores in the soil. The connections between the soil particles became tighter, thus improving the densification of the soil. The UCS of the RM–MgO and RM–CaO stabilized soil was improved. As can be seen in Figure 2 and Figure 3, with the addition of NaOH, more MgO (or CaO) was consumed for the hydration reaction. This is because that the addition of NaOH provided an alkaline environment, which could facilitate hydration reactions to form CSH [30,32]. Additionally, the cementing effect of CSH was stronger than that of MSH [50], which led to a difference in the enhancement between the RM–MgO and RM–CaO stabilized soil. The reaction process is as follows:

(4) $$\mathrm{MgO}+H_{2}O\to {\mathrm{Mg}\left(\mathrm{OH}\right)}_{2}$$

(5) $${\mathrm{Mg}\left(\mathrm{OH}\right)}_{2}\to {\mathrm{Mg}}^{2+}+2{\mathrm{OH}}^{-}$$

(6) $${\mathrm{Mg}\left(\mathrm{OH}\right)}_{2}+\mathrm{Si}O_{2}+nH_{2}O\to \mathrm{MgO}\cdot \mathrm{Si}O_{2}\cdot (n+1)H_{2}O$$

(7) $$\mathrm{CaO}+H_{2}O\to {\mathrm{Ca}\left(\mathrm{OH}\right)}_{2}$$

(8) $${\mathrm{Ca}\left(\mathrm{OH}\right)}_{2}\to {\mathrm{Ca}}^{2+}+2{\mathrm{OH}}^{-}$$

(9) $${\mathrm{Ca}\left(\mathrm{OH}\right)}_{2}+\mathrm{Si}O_{2}+nH_{2}O\to \mathrm{CaO}\cdot \mathrm{Si}O_{2}\cdot (n+1)H_{2}O$$

## 5. Conclusions

(1) Both MgO and CaO had a good activation effect on the RM. The UCS reached a maximum value of 9.65 MPa when the mass ratio of RM to MgO was 3:4 and a maximum value of 7.48 MPa when the mass ratio of RM to CaO was 3:2.

(2) The UCS of the RM–MgO (or RM–CaO) stabilized soil first increased and then decreased with the decrease in RM content. The UCS reached a maximum value of 1.73 MPa when the mass ratio of the RM–MgO stabilized soil (without NaOH) was 5:5 and a maximum value of 514.95 kPa when the mass ratio of the RM–CaO stabilized soil (without NaOH) was 8:2. Compared with the RM–MgO stabilized soil after adding NaOH, the enhancement of the UCS of the RM–CaO stabilized soil by NaOH was much greater. The optimum ratios for the RM–MgO stabilized soil (with NaOH) and the RM–CaO stabilized soil (with NaOH) were 2:8 (1.87 MPa) and 4:6 (2.32 MPa), respectively. The stabilized soil’s UCS values are, in increasing order, as follows: the RM–CaO stabilized soil (without NaOH); RM–MgO stabilized soil (without NaOH); RM–MgO stabilized soil (with NaOH); and RM–CaO stabilized soil (with NaOH).

(3) The ability of the stabilized soil to resist freeze–thaw erosion was significantly improved by adding the RM. With the addition of the NaOH solution, the mass loss rate, volume expansion rate, and UCS reduction in the RM–MgO (or RM–CaO) stabilized soil after the freeze–thaw cycles were significantly lower than those of the pure CaO or pure MgO stabilized soil. The RM–MgO stabilized soil showed better resistance to mass loss and volume expansion than the RM–CaO stabilized soil.

(4) The main reason for the elevated UCS of the RM–MgO (or RM–CaO) stabilized soil was the formation of cementitious materials, such as CSH and MSH. Freeze–thaw cycles did not weaken the formation of its CSH and MSH but caused the pore volume of the specimen to become larger and cracks to form inside the specimen. The decrease in the UCS of the specimen was mainly due to these physical damages.

The test method for the determination of the California bearing ratio, immediate bearing index, and linear swelling was not considered in this study. If red mud is utilized in road base materials, these tests should be considered. So, in our next study, we will consider these tests.

## Figures and Tables

**Table 1:** Chemical composition of soil and binders (% by weight).

Composition	CaO	SiO_2_	Fe_2_O_3_	Al_2_O_3_	MgO	Na_2_O	Others	Loss on Ignition
Kaolin	0.06	53.59	0.89	43.24	0.05	0.07	2.10	0
RM	8.22	27.33	26.08	20.95	1.23	9.61	6.58	0
MgO	5.62	10.23	0.03	6.34	76.72	0.27	0.79	0
CaO	92.58	1.87	0.36	1.30	3.10	0.15	0.62	0.02

**Table 2:** Testing program for RM-based cementitious material.

Test Group No.	Water–Binder Ratio	RM (%)	MgO (%)	CaO (%)	Days of Curing
M-1	0.5	75	25	0	7, 14, 28
M-2		60	40	0	
M-3		43	57	0	
M-4		33	67	0	
L-1		75	0	25	
L-2		60	0	40	
L-3		50	0	50	
L-4		43	0	57	

**Table 3:** Design of mixing proportions.

Test Group No.	RM (%)	MgO (%)	CaO (%)	NaOH (mol/L)	Days of Curing
RM-1	90	10	0	0	7, 14, 28
RM-2	80	20	0	0	
RM-3	70	30	0	0	
RM-4	60	40	0	0	
RM-5	50	50	0	0	
RM-6	40	60	0	0	
RM-7	30	70	0	0	
NRM-1	90	10	0	1.25	
NRM-2	80	20	0	1.25	
NRM-3	70	30	0	1.25	
NRM-4	60	40	0	1.25	
NRM-5	50	50	0	1.25	
NRM-6	40	60	0	1.25	
NRM-7	30	70	0	1.25	
NRM-8	20	80	0	1.25	
NRM-9	10	90	0	1.25	
NRM-0	0	100	0	1.25	
RL-1	90	0	10	0	
RL-2	80	0	20	0	
RL-3	70	0	30	0	
RL-4	60	0	40	0	
RL-5	50	0	50	0	
NRL-1	90	0	10	1.25	
NRL-2	80	0	20	1.25	
NRL-3	70	0	30	1.25	
NRL-4	60	0	40	1.25	
NRL-5	50	0	50	1.25	
NRL-6	40	0	60	1.25	
NRL-7	30	0	70	1.25	
NRL-0	0	0	100	1.25	

## Data Availability

All data, models, and code generated or used during this study appear in the submitted article.
